# Correction: Role of polysilicon in poly-Si/SiO_*x*_ passivating contacts for high-efficiency silicon solar cells

**DOI:** 10.1039/d0ra90049d

**Published:** 2020-05-06

**Authors:** HyunJung Park, Soohyun Bae, Se Jin Park, Ji Yeon Hyun, Chang Hyun Lee, Dongjin Choi, Dongkyun Kang, Hyebin Han, Yoonmook Kang, Hae-Seok Lee, Donghwan Kim

**Affiliations:** Department of Materials Science and Engineering, Korea University Seoul 02841 Republic of Korea; KU-KIST Green School Graduate School of Energy and Environment, Korea University Seoul 02841 Republic of Korea ddang@korea.ac.kr lhseok@korea.ac.kr solar@korea.ac.kr

## Abstract

Correction for ‘Role of polysilicon in poly-Si/SiO_*x*_ passivating contacts for high-efficiency silicon solar cells’ by HyunJung Park *et al.*, *RSC Adv.*, 2019, **9**, 23261–23266. DOI: 10.1039/c9ra03560e

The authors regret that the list of corresponding authors was incorrect in the original article. The corrected author list and associated contact details are as shown above.

The Royal Society of Chemistry apologises for these errors and any consequent inconvenience to authors and readers.

## Supplementary Material

